# Associations of City-Level Active Aging and Age Friendliness with Well-Being among Older Adults Aged 55 and Over in Taiwan

**DOI:** 10.3390/ijerph17124304

**Published:** 2020-06-16

**Authors:** Hui-Chuan Hsu

**Affiliations:** School of Public Health, Research Center of Health Equity, College of Public Health, Taipei Medical University, Taipei 11031, Taiwan; gingerhsu@tmu.edu.tw

**Keywords:** active aging, age-friendly city, health-related quality of life (HRQoL), well-being, life satisfaction, older adults

## Abstract

This study aims to identify the typology of city-level active aging and age-friendliness across cities in Taiwan and examine their effects on well-being in terms of life satisfaction (aged 55 and over) and health-related quality of life (HRQoL) (aged 65 and over) among older adults. The data were from the 2017 Taiwan Senior Citizen Condition Survey. Available indicators of Taiwan’s Active Aging Index and city age-friendliness were selected, and mixed linear models were analyzed. Active aging cities were classified into four categories—*content, developed, participatory,* and *pioneer*—and age-friendly cities into *insecure*, *infrastructural*, and *tranquil*. Life satisfaction was rated higher in *content* and *participatory* cities compared with *the pioneer* city, and related to individuals’ active aging status. Physical HRQoL was rated higher in *infrastructural* and *tranquil* cities, compared with *insecure* cities. City types of active aging and age-friendliness have different effects on well-being, but the effects are weaker than those of individuals’ characteristics.

## 1. Introduction

Active aging has become the new paradigm for policymakers in aging societies along with the global population’s aging trend. Active aging was first defined by the World Health Organization [1] as “the process of optimizing opportunities for health, participation and security in order to enhance quality of life as people age”. Later, to describe countries’ active aging status for the European Union (EU), the United Nations Economic Commission for Europe [2] proposed the Active Aging Index (AAI), comprising four domains of 22 indicators: employment; participation in society; independent, healthy, and secure living; and the capacity for active aging and a supportive environment. The EU’s AAI score was found to be positively related to the gross domestic product and life satisfaction [3]. The AAI has been applied not only at the country level, but also at the region and city levels [4,5]. Whether AAI is applied at the country or city level, policies regarding active aging can be conducted at the city level, and local governments are responsible for implementing active aging policies. However, differences in active aging are often found between regions and cities, including Taiwan [6]. According to WHO’s active aging framework [1], environmental factors are determinants to individual’s active aging. Meanwhile, the concept of age-friendly cities, as suggested by WHO [7], has been implemented around the world, using active aging as a guide: “In an age-friendly city, policies, services, settings and structures support and enable people to age actively”. WHO’s description of an of age-friendly city involves eight domains: housing, transportation, outdoor spaces and buildings, community support and health services, communication and information, social participation, civic participation and employment, and respect and social inclusion. Therefore, city-level factors such as a supportive environment can affect an individual’s active aging status. 

The purpose of this study is to examine the association between an individual’s active aging status and city-level active aging and age-friendliness in Taiwan. This study focuses on city-level differences in terms of the city types of multiple indicators and the effects of city-level types on older adults’ well-being.

### 1.1. Active Aging and Age Friendly City

Active aging and age friendly environment are policy frameworks promoted in the recent decades. The concept of active aging may be related to the classic gerontology theories: activity theory or continuity theory [8] and successful aging [9]. WHO proposes the Active Aging policy framework in 2002 [1] and suggest three domains of active aging: health, security, and participation. When EU suggest Active Aging Index in 2012, the index has been widely applied in the studies globally in recent years, and the performance of Active Aging Index was found to be positively related to gross domestic product (GDP) and quality of life [5,10], but not related to population aging level [11]. Some of the Active Aging Index indicators are not suitable for Asian culture, and the indicators culture should be tailored for different culture [6].

Age friendly city is also promoted by WHO [7]. WHO’s active aging policy framework [1] indicates that the following factors can affect active aging: behavioral factors, personal characteristics, social factors, economic factors, health and social services, the physical environment, and gender and culture. The context provides people with opportunities in terms of quality of life in old age, and environmental influences can affect an individual’s behaviors and values regarding well-being. Thus, age friendly city concept is rooted on the active aging concept but more focus on the environmental perspective. The principle of age friendly city takes a bottom-up participatory approach to deal with the age friendly environment issues [7]. Experience of building up an age friendly city or are widely discussed [12,13,14,15,16]. Taiwan also encourages local government to build up age friendly cities as a health policy [15]. The measures for age friendliness may use city-level indicators, or assess individuals’ perception by a scale [17]. 

### 1.2. Theoretical Explanations of Contextual Influence

The ecological perspective has been applied in gerontology since 1960s [18]. Bronfenbrenner [19] suggests an ecological system perspective to examine human behavior and development, explained by individual factors (microsystem), interpersonal relationships (mesosytem), the external environment (exosystem), social values and beliefs (macrosystem), and life course transition (ecological transition). It is reasonable to assume that environmental factors at different levels can affect active aging, including city-level factors. City-level factors consist of not only the infrastructure and physical environment, but also interpersonal interactions in neighborhoods, community resources, social capital, health and community services and policies, economic development, and lifestyles. 

Although Active Aging Index is designed for a nation or a city as the policy tool, researchers also use active aging indicators at the individual-level [20,21]. However, the effect of both the city/community level and individual level of active aging to individual’s well-being is little considered. Age friendly environment (cities or communities) is often discussed as the contextual factors in the research as the following literature review. 

### 1.3. Older People’s Well-Being: Individual- and Community-Level Factors and Age Friendly Environment

There are different definitions of well-being, happiness, or quality of life [22,23]. In this study, well-being is viewed from a cognitive and subjective perspective, in terms of life satisfaction and quality of life. This study focuses on health-related quality of life (HRQoL), in support of the goal of health and well-being underlying the concepts of WHO’s active aging and age-friendly cities. Individual factors related to life satisfaction or HRQoL have been widely explored among older adults. Life satisfaction and HRQoL are related to age, gender, personal values, awareness of age-related change, demographic characteristics, socioeconomic status, mental health, living arrangements, health conditions, resilience, social support or social connectedness, social participation, absence of material deprivation, home ownership, years of residence, ability to drive, and so on [24,25,26,27,28,29,30,31,32]. These indicators are closely related or similar to active aging indicators. Empirical studies find neighborhood attributes to be related to individuals’ well-being. The community or neighborhood characteristics related to well-being or quality of life in these studies include aggregated characteristics (e.g., education, income), the social environment (e.g., security, solidarity or social cohesion, and neighborhood social capital or social trust), and the built environment or infrastructure (e.g., neighborhood service accessibility, walking areas, and aesthetics) [28,33,34,35]. Although active aging indicators have been applied at the city or regional level to gauge the area’s active aging status [4,5], the relation between area-level active aging and well-being has not been examined. 

Age-friendly communities also have effects on well-being. An age-friendly environment is positively related to life satisfaction, self-perceived health [29,30,36,37,38,39], successful aging [40], and life satisfaction [41]. Park and Lee [38] find that an age-friendly environment (in terms of housing, transportation, neighborhood, the service environment, social participation, and social inclusion) is related to higher life satisfaction among older South Koreans, especially for those who are living alone and in poverty. Additionally, age friendliness can be more important to life satisfaction for younger elderly cohorts than for older elderly cohort [29], and different age groups can have different demands in terms of neighborhood environments [42]. A more age-friendly environment can thus offset an individual’s disadvantages and promote psychological well-being. A study in Hong Kong found that the domains related to life satisfaction between young-old and old-old are not the consistent; the common domains of age friendliness to life satisfaction for two generations are transportation and social participation [43], and sense of community may be the mediating factor between age friendly perception and life satisfaction [44]. 

### 1.4. Older People’s Active Aging: Individual- and Community-Level Factors and Age Friendly Environment

Lak et al. [39] conduct a systematic review of the literature to examine the effects of cities and the environment on older adults’ active aging. They present five themes (including 15 subthemes) of active aging extracted from the literature, which they label: Persons (persons characteristics and behavioral attitudes), place (land-use, access, physical form, cityscape/city image, public open spaces, and housing), process (social environment, cultural environment, economic environment), policy making (good governance), and prime (physical health, mental health, and social health). This ecological framework suggests a multilevel and multi-domain perspective for promoting active aging. Older people’s individual characteristics are found to be related to active aging, including age (being younger), gender, higher education, absence of disability, higher income, absence of material deprivation, and engagement with the community [45,46]. Community-level factors related to active aging include a higher percentage of college-educated residents, a higher percentage of migrant residents, more urban development, sufficient infrastructure, and greater community capacity in terms of shared interests and collaboration [45,46]. Empirical studies show that an age-friendly environment is positively related to an individual’s active aging [38].

### 1.5. Background

Taiwan is an island in East Asia. The ancestors of the Taiwanese came from mainland China. Taiwan was colonized by Japan from 1895 to 1945, during which period agriculture, health care, and education systems were established. After the Chinese Civil War, in 1949, many soldiers and families emigrated from mainland China to Taiwan. Taiwan experienced dramatic economic growth after the 1960s and became one of the Four Asian Tigers during the 1960s through the 1970s. By the time martial law was lifted in 1987, Taiwan’s political system had become a democracy. The traditional culture of the Taiwanese originated mainly in China, where Confucian culture emphasizes filial piety and respect toward elders. The family-centered culture of the Taiwanese expects people to care for and support their elderly parents. However, under Westernization and modernization, the social values of aging and aging care have been gradually changing to those of developed Western countries. Taiwan has a population of 23.58 million [47], with a gross domestic product per capita of U.S. $27,298 [48]. Taiwan has one of the world’s fastest-aging societies, with the percentage of the elderly (aged 65 and over) in the population increasing from 7% in 1993 to 14.56% in 2018 [47]. In addition to national health insurance and long-term care policies, active aging and age-friendly cities are becoming core elements of the aging policies of the Taiwanese Ministry of Health and Welfare’s Health Promotion Administration, especially age friendly city programs are widely encouraged in all the cities in Taiwan [15]. Taiwan’s AAI (TAAI) is based on the EU’s AAI [6], and a Taiwanese age-friendly city index has been suggested [49]. Based on 2017 data, differences in the Taiwanese sample across rural and urban areas have been identified [50]: Active aging indicators related to infrastructure and economic status are better in urban areas, whereas indicators related to physical activity, mental well-being, and social inclusion are better in rural areas. Unfortunately, the sample is not representative of the nation in terms of covering all cities in Taiwan. City differences in age friendliness are also found [49], but there is no systematic trend. This implies that active aging and age friendliness, as a multi-domain and multi-discipline concept, exhibit unique strengths and weaknesses across cities. However, whether such complicated combinations of multiple indicators can be viewed from a systematic perspective to provide information for the policymakers of cities and the central government is not known. Furthermore, the typologies of active aging for individuals were identified in previous research [51], but the typologies of cities in active aging and age friendliness have not been explored in the existing research.

Therefore, this study aims to identify the typology of city-level active aging and age friendliness across all cities in Taiwan and to examine their effects on well-being in terms of life satisfaction and HRQOL among older adults.

## 2. Materials and Methods 

### 2.1. Data and Sample

This cross-sectional study linked individual- and city-level data. The individual data were from the 2017 Taiwan Senior Citizen Condition Survey, conducted through face-to-face interviews by the Ministry of Health and Welfare. The sample comprised individuals aged 55 years and older, including a community-based sample and an institutional sample. The community-based sampling frame was based on the household registration system and includes those aged 55 years and older, living in Taiwan or the island areas. Due to the policy demands, the sampling for the age 55 to 64 population and the age 65 years old or more population were separated (2491 persons of the age 55–64 group and 3732 persons of the age 65+ group were selected as the sample), and then the total sample was weighted according to the population. The sampling was drawn for each city/county and by town/district and persons based on the age distribution. The samples are thus representative of the age distribution of each city/county. The institutional sampling frame was based on actual residents aged 55 years and older, living in long-term care institutions, veterans housing, nursing homes, senior housing, and senior apartments. The stratified sampling for the institutional sample was by city/county and by institution, and a systematic random sampling of three persons per institution was used to obtain the sample for each institution. There were 647 persons selected as the institutional sample. If the sample refuse to do the interview or did not complete the interview, the replacing sample would be used. The completed community-based sample was 6238 persons (with completion rate of 44.9% of the contacts), and the completion rate of the contacts of the institution-based sample was 682 participants (with completion rate of 91.4%). In total 6920 participants were included in this study. The characteristics of the completed sample was not significantly different from the demographic distribution of the population by the test goodness of fit. 

Two kinds of questionnaires were designed: one for those aged 55–64 and one for those aged 65 and older. Participants who were unable to communicate (e.g., due to frailty, mental condition, a hearing problem) or unable to finish the whole interview could use a proxy (a family caregiver or relative/friend) to answer the survey. The Taiwan Senior Citizen Condition Survey provided the individual-level and aggregated city-level active aging data for this study. Data on a total of 20 cities/counties were used (combining the islands of Kingmen and Lienchiang as one group). Other city-level indicators were obtained from data from the 2017 survey on the Monitoring of Age Friendly Cities in Taiwan [49]. The current study obtained the approval of the Taipei Medical University Joint Institutional Review Board (No.201907022) before starting.

### 2.2. Measures

#### 2.2.1. Individual-Level Data

1. Life satisfaction: Life satisfaction was scored from one to five, to indicate very unsatisfactory to very satisfactory, respectively (score 1–5).
2. HRQoL: The HRQoL variable was measured based on the 12-Item Short-Form Health Survey HRQoL [52]. Only six variables were covered in the survey. The variables for the physical domain of HRQOL were self-rated health, physical role limitations, and difficulty carrying groceries. The variables for the mental domain of HRQoL were social role limitations, feeling energetic, and feeling depressed. The scoring for each variable was converted to a value of zero to 100, with a higher score representing better health. Thus, the total HRQOL score ranged from zero to 600, with physical HRQOL and mental HRQOL scored of zero to 300 each. The HRQOL variables were only available for the questionnaire for those aged 65 and older.

The active aging indicators were based on the EU’s AAI and the Taiwan’s Active Aging Index (TAAI) [6]. The indicators selected according to the four domains of the TAAI are as follows.

1. Employment: Work (yes/no).
2. Social participation: Social participation included volunteering, political participation, family caregiving (children or older family members), and participating in other social group. Each variable was scored yes or no.
3. Independent, healthy, and secure living: Physical function independence was measured as having no difficulties or only one item of difficulty in activities of daily living (eating, dressing, transferring, walking indoors, going to the bathroom, bathing). Living independently was defined as living alone or only with a spouse. The financial security variables included home ownership (yes/no) and non-poverty (i.e., a personal income of more than U.S. $600 per month).
4. Capacity and supportive environment: Social connection was defined as contact with family or friends at least once a week. Use of information and communications technology (ICT) was defined as using the Internet at least once a week. Mental well-being was defined by not being depressed (yes/no) used for life satisfaction models, and happiness item (yes/no) used for HRQOL models. Lifelong learning was measured by participation in any kind of education program. The public transportation convenience and safety items were coded as either inconvenient, convenient, or not used. Elderly respect was rated as whether the public respects the elderly or not. The control variables for the participants include age (ages 55–59, 60–64, 65–69, 70–74, and 75 and above), gender, education (ordinal), marital status (married or not), and self-rated health (with a score of one to five).

#### 2.2.2. City-Level Data

The city-level active aging indicators were the same as for the individuals, but aggregated by city. Higher education (the percentage of the residents whose educational level was high school or above) was also included in the capacity and supportive environment domain of city-level indicators. In total, 17 active aging city (AAC) indicators are used to form the AAC clusters among the 20 cities. The indicators are selected based on WHO’s [7] age-friendly city framework. Age-friendly city (AFC) indicators were selected referring to previous research [49,53]. The 2017 AFC data were from the report of Taiwan’s age-friendly city indicators [49], or aggregated from the 2017 Taiwan Senior Citizen Condition Survey. The AFC indicators were coded as city rankings in the following:

1. Housing: Housing affordability rate.
2. Outdoor spaces and buildings: Rate of barrier-free public buildings, rate of barrier-free pathways outside the home, the crime rate, and the neighborhood safety rating.
3. Community support and health services: number of medical professionals per population, number of hospital beds per population, and numbers of public libraries.
4. Communication and information: Internet usage rate and lifelong learning rate.
5. Civic participation and employment: Work rate.
6. Social participation: Social group participation rate, volunteer rate, and family caregiving rate.
7. Respect and social inclusion: Elderly abuse rate, social connection rate, low-income people percentage of the population, proportion of the elderly who felt respected.

### 2.3. Analysis

The analysis was conducted by IBM SPSS software version 22.0 (IBM, SPSS Inc., Chicago, IL, USA). Descriptive analysis and one-way analysis of variance (ANOVA) by city/county were conducted first. Because active aging and age-friendly cities were defined by multiple indicators, cluster analysis was applied to classify cities by their active aging and age-friendly status. Hierarchical clustering was conducted first to observe reasonable groups, and K-means clustering was applied to determine AAC city clusters and AFC city clusters. The characteristics of the clusters were accordingly used to name the types of AAC or AFC city clusters. Next, a mixed linear model was applied to examine the effects of city- and individual-level variables on the outcome variables. Self-rated health was only used for the life satisfaction model to avoid redundancy with HRQoL items, and depression was replaced by happiness in measuring mental well-being in the HRQoL models. 

## 3. Results

**Figure 1:**
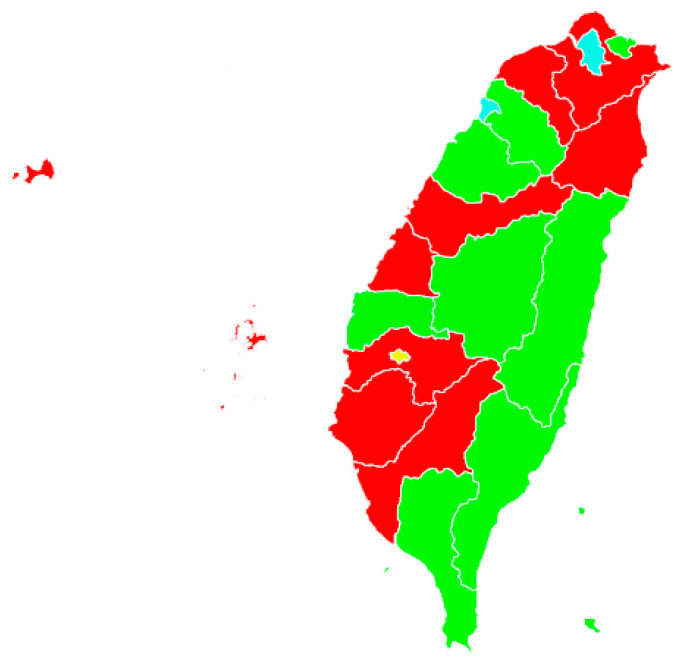
Map of active aging city clusters in Taiwan (Note: Green: Participatory, Light-blue: Developed; Red: Content; Yellow: Pioneer).

Table 1 describes the sample, with both unweighted and weighted percentages. The sample was only weighted when aggregated city indicators were calculated; otherwise, the unweighted sample was analyzed. The sample size for each city are shown in the Supplement Table S1. Table 2 shows the results of the cluster analysis of the active aging indicators (AAC) for the 20 cities. Four active aging city clusters are identified, as shown in Figure 1: Nine cities, or 65.9% of the sample, within the *content* cluster; two cities, or 13.4%, within the *developed* cluster; eight cities, or 19.0%, within the *participatory* cluster; and one city, or 1.7% of the sample, within the *pioneer* cluster. The AAC clusters were named according to the characteristics in Table 2. Cities within the first cluster exhibited a moderate performance in most of the indicators. The cluster had lower levels in social group participation, economic security, public transportation convenience, and elderly respect, but individuals in these cities include the highest percentage of non-depressed persons. Thus, this AAC cluster was named as the *content* cluster. The second cluster was named as *developed*, because cities in this cluster showed low levels of social participation, but high scores for Internet use, public transportation convenience and highly education. Cities within the *participatory* cluster exhibited the highest levels of work, volunteering, family caregiving, and social group participation, which are employment and social participation domains in Active Aging Index. The *participatory* cluster also showed high physical independence, home ownership, and social connections, but low levels of education, ICT usage, and respect for the elderly. The fourth cluster (only one city), was the first city to join the Age Friendly City Program in Taiwan [15], named as the “pioneer” cluster. The *pioneer* city had the high levels of work, volunteering, caregiving, social group participation, economic security, physical independence, lifelong learning, transportation convenience, and safety, but scored lower in terms of mental well-being and social connections. It seems this city showed a strong ability in the three domains of active aging, i.e., employment, social participation, and independent, secure, and healthy living; but the city exhibited a lower well-being and social connectedness, that indicated a lower performance in the domain of capacity and supportive environment in active aging. 

**Figure 2:**
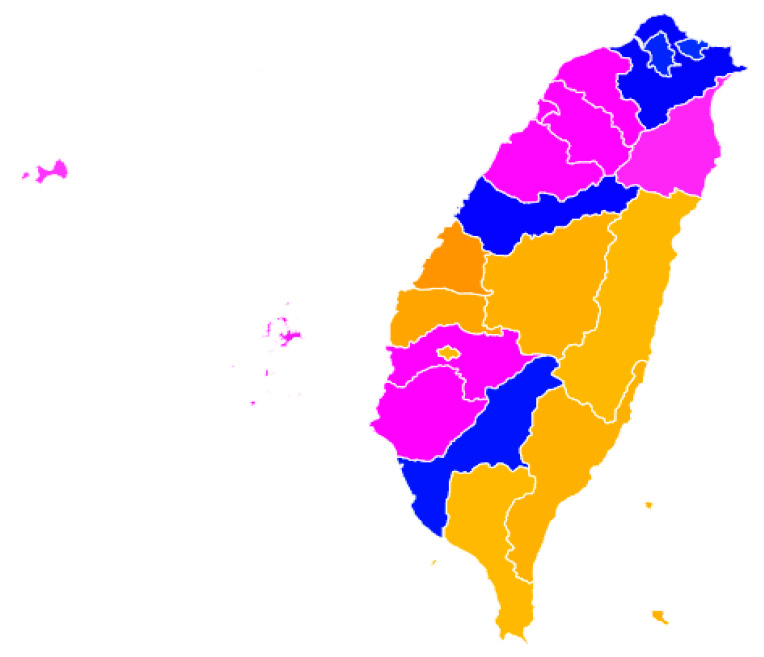
Map of age friendly city clusters in Taiwan (Note: Blue: Infrastructural, Pink: Tranquil, Orange: Insecure).

Cluster analysis was also carried out on the age-friendly city indicators, and the results are shown in Table 3 and Figure 2. Three clusters were identified. Cities in the *insecure* cluster (seven cities, or 19.9% of the sample) scored higher in terms of work, social participation (volunteering, social group participation, and caregiving), social connection, affordable housing, and barrier-free buildings and outside pathways, but poorer regarding barrier-free sidewalks, transportation convenience, crime rate and neighborhood safety. This city cluster was less developed in the security-related domains of age friendly environment outside households. Thus, this AFC cluster was named as the *insecure* cluster. Cities in the *infrastructural* cluster (five cities, or 50.7% of the sample) scored higher in terms of medical resources, barrier-free sidewalks, and Internet use, public libraries, and low-income rate, but low in work, some kinds of social participation (volunteer and caregiving), and housing affordability. It seems the infrastructure for age friendly environment was built well in this cluster, but there the high low-income rate and very low housing affordability may indicate an economic inequality. Cities in the *tranquil* cluster (eight cities, or 29.4% of the sample) scored lower in terms of connections (social group participation, social connections, and Internet use), development (medical resources, public transportation, and barrier-free pathways), but high in terms of elderly respect and neighborhood safety. 

**Figure 3:**
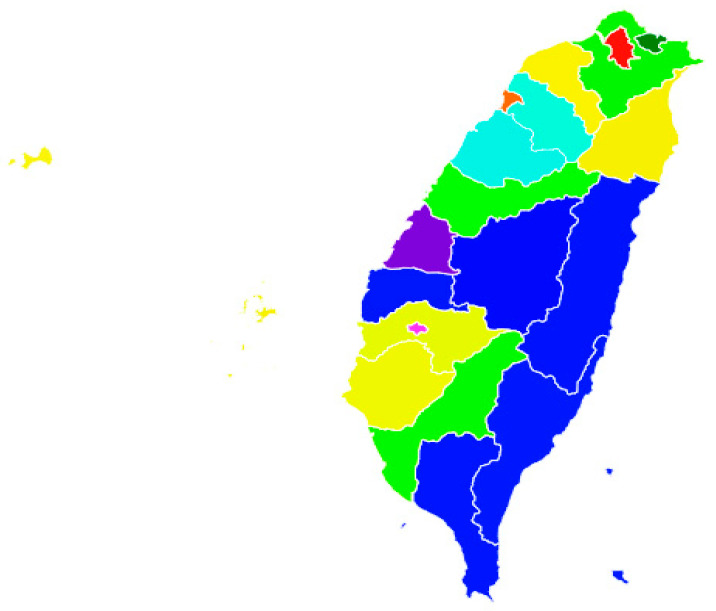
Map of active aging and age friendly city clusters in Taiwan. Note: The city clusters color (city numbers): Developed and Infrastructural: red (1); Developed and Tranquil: orange (1); Content and Tranquil: yellow (5); Content and Infrastructural: light green (3); Participatory and Infrastructural; dark green (1); Participatory and Tranquil: light blue (6); Content and Insecure: dark purple (1); Participatory and Insecure: blue (5); Pioneer and Insecure: pink purple (1).

There were in total nine combinations of the AAC and AFC clusters, which are shown in Figure 3. Among the 12 possible combinations, there were 9 combinations found in this study. Although some of the indicators were overlapping in age friendliness and active aging, the correlations of these two concepts were not necessarily consistent. The combinations and the city numbers are as following: Developed and Infrastructural: (1); Developed and Tranquil (1); Content and Tranquil (5); Content and Infrastructural (3); Participatory and Infrastructural (1); Participatory and Tranquil (6); Content and Insecure (1); Participatory and Insecure (5); Pioneer and Insecure (1). 

Life satisfaction and HRQoL across the AAC and AFC clusters were compared by one-way ANOVA (see Supplement Figure S1). There were significant differences between the AAC clusters for life satisfaction and HRQoL: *developed* cities showed the highest levels of life satisfaction, overall HRQoL, and physical HRQoL, while content cities exhibited the highest mental HRQoL levels. Pioneer cities showed the lowest levels of life satisfaction and HRQoL. There were also differences in the AFC clusters between life satisfaction and HRQoL: tranquil cities showed the highest level of life satisfaction and mental HRQoL, but infrastructural cities showed the highest overall HRQoLand physical HRQoL levels. Insecure cities exhibited the lowest levels of both life satisfaction and HRQoL.

Mixed linear modeling was then applied to examine the associations of the clusters and individual active aging status of city with life satisfaction (Table 4) and with HRQoL (Table 5). In Table 4, the pioneer AAC city and insecure AFC cities were used as the reference groups. AAC cities were significantly related to life satisfaction: individuals living in so-called *content* and *participatory* cities had higher levels of life satisfaction than those living in *pioneer* city. The AFC clusters were not significantly different. Higher life satisfaction was related to individual’s greater age, being female, higher education, better self-rated health, not being a family caregiver, physical independence, not being depressed, living with others, home ownership, non-poverty, socially connectedness, Internet use, lifelong learning, and a high elderly respect score.

Table 5 shows the association between HRQoL for adults aged 65 and older with city and individual factors. The AAC and AFC clusters were not significant in the overall HRQoL. Higher HRQoL was noted among older adults who were younger, male, working, volunteering, no political participation, physically independent, happy, socially connected, using the Internet, rating public transportation as safe, and feeling the elderly as being respected. AFC clusters were significantly related to the physical domain of HRQoL: older adults living in infrastructural and tranquil cities show higher physical HRQoL levels, as do those who were younger, working, volunteering, physically independent, happy, and socially connected and who used the Internet. Regarding the mental domain of HRQoL, AAC and AFC were not significant. Higher mental HRQoL was associated with those who were younger, married, working, volunteering, physically independent, happy, living with others, who did not engage with social groups or politics, did not provide family care, own their home, not poor, socially connected, use the Internet, did not use public transportation, rated transportation as safe and felt the elderly were respected.

## 4. Discussion

This study first used indicators of active aging and age friendliness to identify the types of cities and then examines the effects of individuals’ active aging status and city-level active aging and age friendliness on older people’s levels of life satisfaction and HRQoL. Four types of active aging cities (AAC) are identified: *content, developed, participatory,* and *pioneer*. Three types of age-friendly cities (AFC) are identified: *insecure, infrastructural*, and *tranquil*. Generally, individuals’ active aging status showed a stronger effect on life satisfaction and HRQoL than the type of city. The level of life satisfaction of the participants aged 55 years and older was related to AAC and the individual’s age, gender, education, self-rated health, family caregiving, physical function, depression, living arrangement, financial status, social connectedness, use of ICT, lifelong learning, and respect for the elderly; the effect of AFC was not significant. The overall HRQoL of the participants aged 65 and older was related to the individuals’ age, gender, work, different social participations, physical function, mental well-being, social connectedness, use of ICT, public transportation safety, and respect for the elderly; HRQoL was not significantly related to either AAC or AFC. Regarding HRQoL, *infrastructural* and *tranquil* AFCs had a stronger relation with physical HRQoL, and AAC and AFC were not significantly related to mental HRQoL.

### 4.1. City Typology and Indicators of Active Aging and Age Friendliness

City-level active aging and age friendliness have been measured in previous research [4,29,30,36,37,38,40,41]. Other community- and neighborhood-level indicators, such as built environment and infrastructure, the social environment, and aggregated population characteristics, have also been applied to describe the characteristics of cities [18,33,34,35,37,45,46]. However, these indicators could overlap, and the different indicators of active aging or age friendliness might therefore not show consistent effects on well-being. Although the components of active aging and age friendly city have been recognized, multiple domains should be considered at the same time to exhibit the unique strengths and weakness of a city. In addition, association of individual’s perception of age friendliness and active aging has been explored [37]; however, this current study is the first to identify the city types of multiple indicators of active aging and age friendliness simultaneously. To view the multiple domains of active aging and age friendliness of a city may provide an in-depth qualitative description based on the multiple quantitative indicators. The data-driven results may not be directly compared across countries, but such approach would show the distinguishing features of the cities, and then the policies or local strategies may be accordingly developed. 

### 4.2. City Typology and Well-Being

In this study, the active aging status of AAC were identified as either *content*, *developed, participatory*, or *pioneer*. The most interesting type was *content* cities, because their active aging indicators were generally rated lower than in most other cities, but their mental well-being was better. The levels of life satisfaction and physical HRQoL of content cities was similar to or only a little lower than in developed cities, but the mental HRQoL level of *content* cities was the highest of all the types. One possible explanation is that the life circle of content cities is affected by nearby cities. A city-based ecological framework cannot measure the effects of cross-city boundaries, and city-level policies do not show consistent effects on all of a city’s individual citizens. Another possible reason is that an individual’s characteristics and active aging performance have a greater effect on well-being, or older adults in content cities have more positive psychological state. Although the city-level typologies are not all significantly related to well-being, when individuals’ active aging and other factors were controlled for, bivariate analyses revealed differences between the city types. In particular, infrastructural and tranquil AFCs showed higher physical HRQoL levels for older individuals. This means that an age-friendlier city can provide an equal chance to older adults of all levels of health and active aging in the physical domain of HRQoL. Not only more developed *infrastructural* urban cities but also low-development *tranquil* cities showed higher physical HRQoL than the *insecure* cities. The infrastructure in *infrastructural* cities could provide a more convenient and accessible environment, while the lifestyle in tranquil cities is calm, elderly inclusive, and healthy, without the chaos and negative effects of urban areas, which is beneficial to the well-being of older adults. This finding reflects the social environment components of age friendliness in previous research [28,36,53]. When policymakers try to improve the infrastructure and resource accessibility of age-friendly cities, they must also prioritize a social atmosphere and culture promoting active aging and age friendliness. In addition, AAC and AFC are not significantly related to individuals’ well-being or mental HROL. In other words, an individual’s active aging is more important for mental well-being than the city environment. This finding implies that if older adults commit to the effort of active aging, they can still achieve a better quality of life and well-being, no matter where they live.

### 4.3. Individuals’ Factors Related to Well-Being

Individuals’ active aging and personal characteristics showed stronger effects on well-being than city-level factors did. More active aging was generally related to higher life satisfaction and HRQoL, as shown in previous research [24,25,26,27,31,32]. However, there were exceptions, such as when caring for a family member or living alone. Although family caregiving is a form of social participation, it also indicates a greater burden on older adults, which can reduce their well-being, consistent with previous research [54]. Public support in long-term care services is therefore needed to reduce the burden of family caregivers. The active aging indicator for living alone independently, as suggested in the AAI, needs to be carefully explained, because it cannot differentiate between willingness or ability to live alone. Additionally, older people in Eastern cultures usually prefer living with family, even if they are healthy and independent [6]. Living alone can also be closely—although not necessarily—related to loneliness and social isolation. Political participation is also negatively related to overall HRQoL and mental HRQoL. Political participation is still low in Taiwan and is found to be beneficial for the maintenance of better cognitive function among the Taiwanese [55]. However, political participation on the Internet has increased [56], and support for different parties has caused arguments in recent years. The impact of the political participation of older adults on mental well-being has not yet been examined. Compared with non-users, public transportation users who rated it as safe enjoy better HRQoL. However, public transportation users, regarding of whether they rated it as convenient, report lower mental HRQoL than non-users. Although it is possible that those who use public transportation are still adapting to the system, non-users (such as older drivers) have greater autonomy in mobility. Public transportation allows access to social participation and thus benefits active aging; however, it can only be instituted by central or local governments, not by the efforts of individuals. Convenient and accessible public transportation for older adults is suggested as a priority of age-friendly cities.

### 4.4. Limitations

This study has limitations. First, all the data were from secondary data. Additionally, some of the variables might not be available, and not all the area-level indicators of age-friendly city or confounders were included in the analysis. Second, the 2017 Taiwan Senior Citizen Condition Survey merges data from the Kingmen and Lienchiang Islands, because of their small numbers. The island group might therefore not be representative of these areas. The sample size for each city may not be big, but this data was the most recent city-representative data containing active aging indicators. Third, since the smallest unit of area-level data was the city, the study assumed that the life circle was confined within the city/county boundaries and was thus unable to estimate the cross-city influences of area-level indicators. Fourth, the city-level data covered the same period as the individual data; therefore, only associations can be determined, and not causal relations. Fifth, due to the limited number of institutional cases, the comparison between community-based and institution-based sample by cities was not conducted. 

## 5. Conclusions

This study identifies city types in terms of active aging and age friendliness and examines individual- and city-level effects on life satisfaction and HRQoL among older adults. Differences between cities are found in multiple domains of active aging and age friendliness, and not only in one direction. By examining multiple domains and multiple indicators of active aging and age friendliness, city typologies can be identified. Understanding the strengths and the weakness of each city is a first step for constructing an active aging or age friendly city, and different strategies to active aging and age friendly cities can then be developed accordingly. Both the physical environment (infrastructure and services) and the social environment are important for well-being. In addition, the variations of cites exists imply social inequality in active aging and age friendliness, that is suggested for further study in the future. The differences between community-based and institution-based older adults in active aging and age friendliness are also suggested to be explored, in order to provide implications and policy suggestion which accommodate all the older adults. The longitudinal effects of city-level characteristics on health and well-being is also suggested for the future studies.

## Figures and Tables

**Table 1:** Description of the sample.

Variables	Unweighted (*n* = 6920) Mean (SD) or %	Weighted (*n* = 6,578,754) Mean (SD) or %
Age	69.25 (9.93)	66.43 (9.19)
Age 55–59	18.6%	27.2%
Age 60–64	19.1%	23.9%
Age 65–69	19.0%	18.2%
Age 70–74	12.4%	10.3%
Age 75–79	12.7%	8.9%
Age 80+	18.2%	11.5%
Sex: Female	52.7%	52.6%
Male	47.3%	47.4%
Residence: Community-based	90.1%	98.8%
Institution-based	9.9%	1.2%
Marital status: No spouse	33.2%	26.1%
Having spouse	66.8%	73.9%
Education (ordinal, 1–22)	5.57 (5.27)	6.06 (5.40)
Self-rated health (1–5)	3.53 (0.94)	3.60 (0.92)
HRQoL (0–600)	468.20 (127.76)	480.12 (120.92)
HRQoL—physical (0–300)	222.08 (73.87)	230.21 (67.57)
HRQoL—mental (0–300)	254.64 (62.64)	260.18 (59.03)
Life satisfaction (1–5)	3.80 (0.82)	3.80 (0.81)
Work	24.8%	31.8%
Volunteering	10.4%	11.5%
Politics	1.6%	1.8%
Other social groups	14.0%	13.9%
Family caregiving (children or elderly)	6.9%	7.8%
Physical independent	93.6%	97.0%
Not depressed	72.8%	75.4%
Happy	84.5%	85.6%
Living alone (or with spouse only)	8.0%	7.3%
Owning house	84.9%	92.8%
Non-poverty	84.9%	26.0%
Social connected	64.0%	68.8%
Use of Internet	35.0%	43.5%
Lifelong learning	5.4%	5.8%
Public transportation: Inconvenient	3.7%	4.0%
Convenient	50.4%	53.4%
Non-users	45.9%	42.7%
Public transportation: Unsafe	1.4%	1.4%
Safe	52.7%	53.4%
Non-users	45.9%	42.7%
Elderly respected	69.5%	68.0%

Note: The weighted sample was weighted by the population distribution.

**Table 2:** City clusters of active aging indicators.

Active Aging Indicators (%)	Content (9 Cities)	Developed (2 Cities)	Participatory (8 Cities)	Pioneer (1 City)
Work	0.306	0.274	0.385	0.369
Volunteer	0.115	0.107	0.132	0.086
Social group participation	0.129	0.178	0.152	0.184
Politics	0.016	0.023	0.027	0.011
Family caregiving	0.077	0.084	0.086	0.085
Physically independent	0.074	0.060	0.088	0.108
Living alone	0.074	0.060	0.088	0.108
Non-depressed	0.773	0.698	0.730	0.632
Owning house	0.932	0.898	0.940	0.835
Non-poverty	0.232	0.274	0.316	0.558
Social connected	0.666	0.670	0.792	0.528
Internet use	0.416	0.537	0.398	0.499
Lifelong learning	0.063	0.043	0.054	0.078
Public transportation convenience	0.958	0.992	0.949	0.992
Transportation safety	0.988	0.978	0.982	1.000
Elderly respect	0.669	0.761	0.669	0.736
Higher educated	0.326	0.592	0.313	0.360

Note: *n* = 20 (cities/counties).

**Table 3:** City clusters of age friendly city indicators.

Age Friendly City Indicators (Ranking) 2016	Insecure	Infrastructural	Tranquil
Work	16.429	6.800	7.625
Volunteer	12.286	8.000	10.500
Social groups	13.286	12.600	6.750
Caregiving	13.000	8.600	9.500
Social connected	13.143	10.800	8.000
Internet use	10.000	14.400	8.500
Lifelong learning	12.286	8.800	10.000
Elderly respect	9.286	9.200	12.375
Sidewalk for people	5.286	12.500	13.813
Medical professionals /population	10.857	13.400	8.375
Barrier-free bus accessibility	14.857	9.200	7.500
Bus stop accessibility	8.429	14.600	9.750
Hospital beds /population	11.143	13.000	8.375
Crime rate	13.286	9.600	8.625
Low income rate	13.286	14.100	5.813
Neighborhood safety	6.000	14.800	11.750
Public library numbers	8.929	15.600	8.688
Barrier-free public buildings	13.000	8.400	9.625
Barrier-free Passage house outside	15.571	8.000	7.625
Housing affordability	13.857	3.600	11.875
Elderly abuse rate	11.857	11.400	8.750

Note: *n* = 20 (city/counties).

**Table 4:** Life satisfaction with city clusters and individual factors of older adults (age 55+).

Variables	M1. City Clusters
**Fixed effects**	
Constant	1.720 (0.146) ***
Age	0.051 (0.007) ***
Sex (male)	−0.073 (0.021) **
Marry (having spouse)	0.018 (0.026)
Education	0.010 (0.002) ***
Self-rated health	0.188 (0.012) ***
AAC Cluster: Content	0.267 (0.104) *
AAC Cluster: Developed	0.162 (0.117)
AAC Cluster: Participatory	0.250 (0.099) *
AFC Cluster: Infrastructural	−0.017 (0.053)
AFC Cluster: Tranquil	0.010 (0.049)
Work	−0.001 (0.024)
Volunteering	0.030 (0.032)
Social groups	−0.028 (0.029)
Politics	−0.016 (0.074)
Caregiving	−0.177 (0.035) ***
Physically independent	0.232 (0.098) *
Not depressed	0.284 (0.023) ***
Living alone	−0.179 (0.039) ***
Owning house	0.243 (0.043) ***
Non-poverty	0.055 (0.024) *
Socially connected	0.118 (0.022) ***
Internet	0.061 (0.024) *
Lifelong learning	0.089 (0.044) *
Public transportation: convenient	−0.073 (0.086)
Public transportation: inconvenient	−0.022 (0.087)
Public Transportation: safe	0.131 (0.084)
Elderly respected	0.253 (0.022) ***
**Random effects covariance of cities**	
Residual	0.549 (0.010) ***
AAC clusters	2.805 × 10^−5^ (0.002)
AFC clusters	0.003 (0.000)
**Model fit**	−2LL = 13,133.028, BIC = 13,159.015

Note: *n* = 5816. Missing cases were excluded listwise. Analysis by mixed linear model. Reference groups of categorical variables: Sex (female), marital status (no spouse), active aging city (AAC) cluster (pioneer), age friendly city (AFC) cluster (insecure), work (no), volunteering (no), social groups (no), politics (no), caregiving (no), physical function (dependent), emotional health (depressed), living arrangement (with others), owing house (no), non-poverty (no, i.e., poor), social connected (no), Internet use (no), lifelong learning (no), public transportation convenience (non-users), transportation safety (unsafe), public transportation (non-user), and respected (no). Order variables: Age (1–6), education (1–22), and self-rated health (1–5). AFC: age friendly city; AAC: active aging city; −2LL = −2log likelihood; BIC = Schwar’z Bayesian Criteria. * *p* < 0.05. ** *p* < 0.01, *** *p* < 0.001.

**Table 5:** Health-related quality of life with city clusters and individual factors of older adults (aged 65+).

Variables	M2. HRQoL	M3. HRQoL-Physical	M4. HRQoL-Mental
**Fixed effects**			
Constant	129.533 (30,167) ***	38.029 (14.142) **	84.927 (15.083) ***
Age	−11.830 (1.712) ***	−7.249 (0.955) ***	−2.922 (0.529) ***
Sex (male)	8.604 (3.888) *	3.248 (2.169)	2.221 (1.533)
Marital status (have spouse)	5.941 (4.528)	3.977 (2.525)	4.611 (1.871) *
Education	0.005 (0.412)	0.051 (0.230)	0.022 (0.158)
AAC Cluster: Content	25.422 (27.938)	12.460 (11.236)	14.147 (14.800)
AAC Cluster: Developed	−7.507 (32.030)	1.989 (12.633)	−2.569 (17.046)
AAC Cluster: Participatory	18.193 (26.038)	10.472 (10.633)	7.998 (13.696)
AFC Cluster: Infrastructural	22.445 (15.671)	12.538 (5.788) *	6.856 (8.577)
AFC Cluster: Tranquil	26.591 (14.160)	12.802 (5.326) *	9.758 (7.706)
Work	31.446 (5.277) ***	18.665 (2.942) ***	11.626 (1.769) ***
Volunteering	29.198 (6.358) ***	14.450 (3.544) ***	11.252 (2.372) ***
Social groups	−4.176 (5.202)	1.873 (2.901)	−7.245 (2.119) **
Politics	−34.220 (13.598) *	−8.756 (7.584)	−16.775 (5.386) **
Caregiving	−8.147 (7.049)	1.045 (3.932)	−6.477 (2.642)*
Physically independent	258.179 (13.555) ***	152.858 (7.561) ***	109.215 (7.100) ***
Happy	33.981 (4.904) ***	12.573 (2.733) ***	21.627 (2.048) ***
Living alone	−7.684 (6.505)	1.043 (3.629)	−8.512 (2.859) **
Owning house	15.986 (8.431)	8.653 (4.701)	7.393 (3.119) *
Non-poverty	−2.310 (4.807)	−3.013 (2.673)	3.672 (1.775) *
Socially connected	30.480 (3.875) ***	14.748 (2.160) ***	13.797 (1.620) ***
Internet use	23.002 (4.767) ***	9.644 (2.653) ***	10.199 (1.787) ***
Lifelong learning	8.379 (8.459)	1.589 (4.719)	4.509 (3.193)
Public transportation: convenient	0.556 (15.003)	12.312 (8.359)	−12.553 (6.297) *
Public transportation: inconvenient	−24.949 (15.101)	−6.796 (8.416)	−17.460 (6.380) **
Public Transportation: safe	39.312 (14.611) **	10.705 (8.150)	25.633 (6.157) ***
Respected	11.381 (3.949) **	2.561 (2.201)	7.182 (1.559) ***
**Random effects covariance**			
Residual	10,541.207 (257.665) ***	3283.654 (80.240) ***	2935.722 (54.675) ***
AAC cluster	29.890 (189.743)	0.897 (22.487)	21.767 (54.529)
AFC cluster	353.006 (0.000)	35.140 (0.000)	107.141 (0.000)
**Model fit**	−2LL = 40,857.346, BIC = 40,881.706	−2LL = 36,925.908, BIC = 36,950.268	−2LL = 62,765.185, BIC = 62,791.171

Note: *n* = 3395. Missing cases were excluded listwise. Analysis by mixed linear model. Reference groups of categorical variables: Sex (male), marital status (no spouse), active aging city (AAC) cluster (pioneer), age friendly city (AFC) cluster (insecure), work (no), volunteering (no), social groups (no), politics (no), caregiving (no), physical function (dependent), mental well-being (not happy),living arrangement (with others), owing house (no), non-poverty (no, i.e., poor), social connected (no), Internet use (no), lifelong learning (no), public transportation convenience (non-users), transportation safety (unsafe), public transportation (non-user), and respected (no). Order variables: Age (1–6), education (1–22). AFC: age friendly city; AAC: active aging city; −2LL = −2log likelihood; BIC = Schwar’z Bayesian Criteria. * *p* < 0.05. ** *p* < 0.01, *** *p* < 0.001.

## Supplementary Materials

The following are available online at https://www.mdpi.com/1660-4601/17/12/4304/s1, Figure S1: Well-being by active aging and age friendly city clusters; Table S1: The sample size and the backgrounds of the cities in 2017; Table S2: Life satisfaction with city and individual factors of older adults (age 55+); Table S3: Quality of life with cities and individual factors of older adults (aged 65+); Table S4: Association of active aging city typologies with individuals’ age, sex, and health by multinomial logistic regression (odds ratios); Table S5: Association of age friendly city typologies with individuals’ age, sex, and health by multinomial logistic regression (odds ratios).
