# Supplementary material for: Associations of City-Level Active Aging and Age Friendliness with Well-Being among Older Adults Aged 55 and Over in Taiwan
Source: Int J Environ Res Public Health. 2020 Jun 16;17(12):4304. doi: 10.3390/ijerph17124304 (PMC7345662; doi:10.3390/ijerph17124304)
Supplement: Supplementary file 1 [file ijerph-17-04304-s001.pdf]

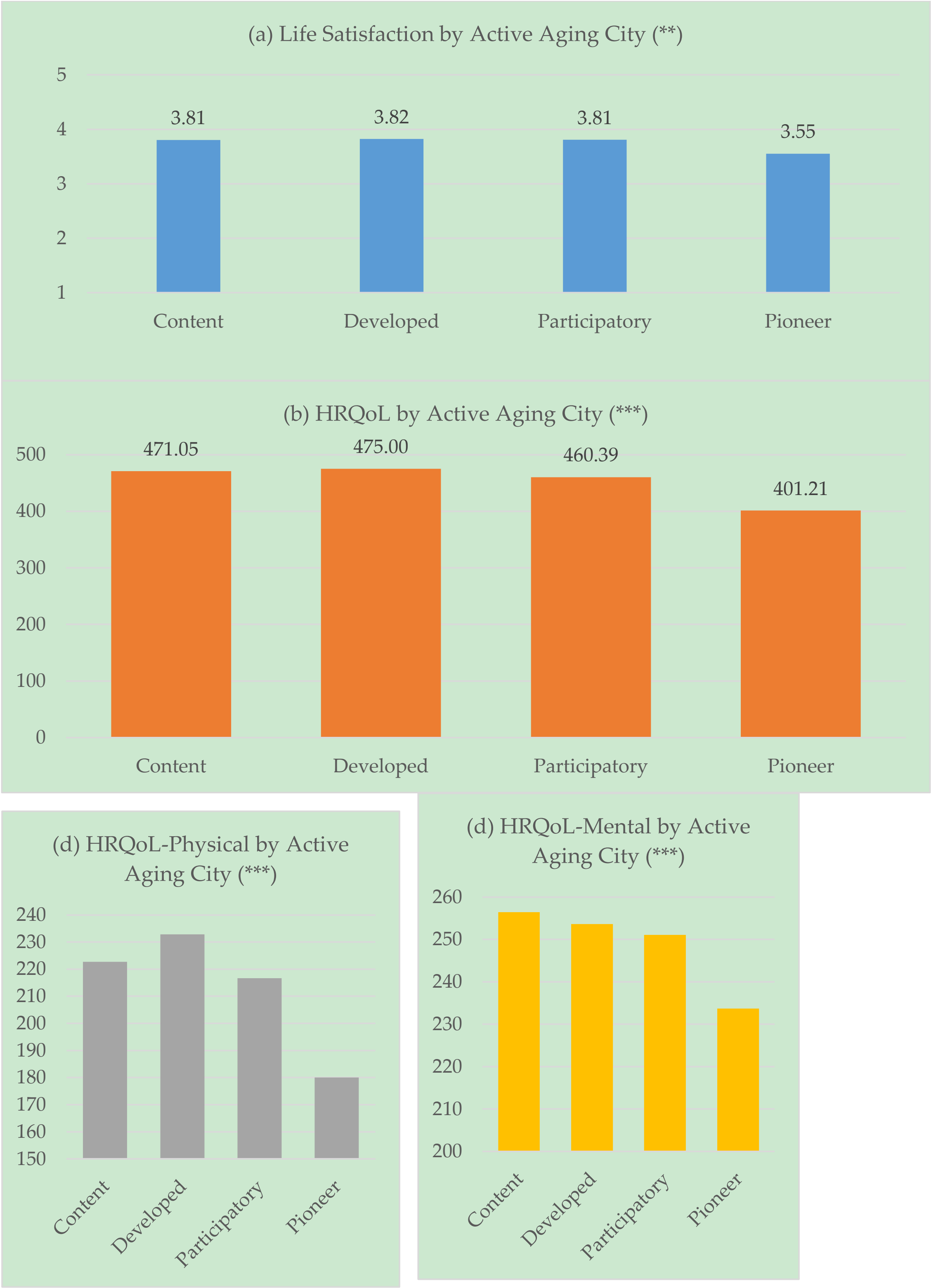
(a) Life Satisfaction by Active Aging City (**)
5
4
3
2
1
3.81
3.82
3.81
3.55
Content
Developed
Participatory
Pioneer
(b) HRQoL by Active Aging City (***)
500
400
300
200
100
0
471.05
475.00
460.39
401.21
Content
Developed
Participatory
Pioneer
(d) HRQoL-Physical by Active Aging City (***)
240
230
220
210
200
190
180
170
160
150
Content
Developed
Participatory
Pioneer
(d) HRQoL-Mental by Active Aging City (***)
260
250
240
230
220
210
200
Content
Developed
Participatory
Pioneer

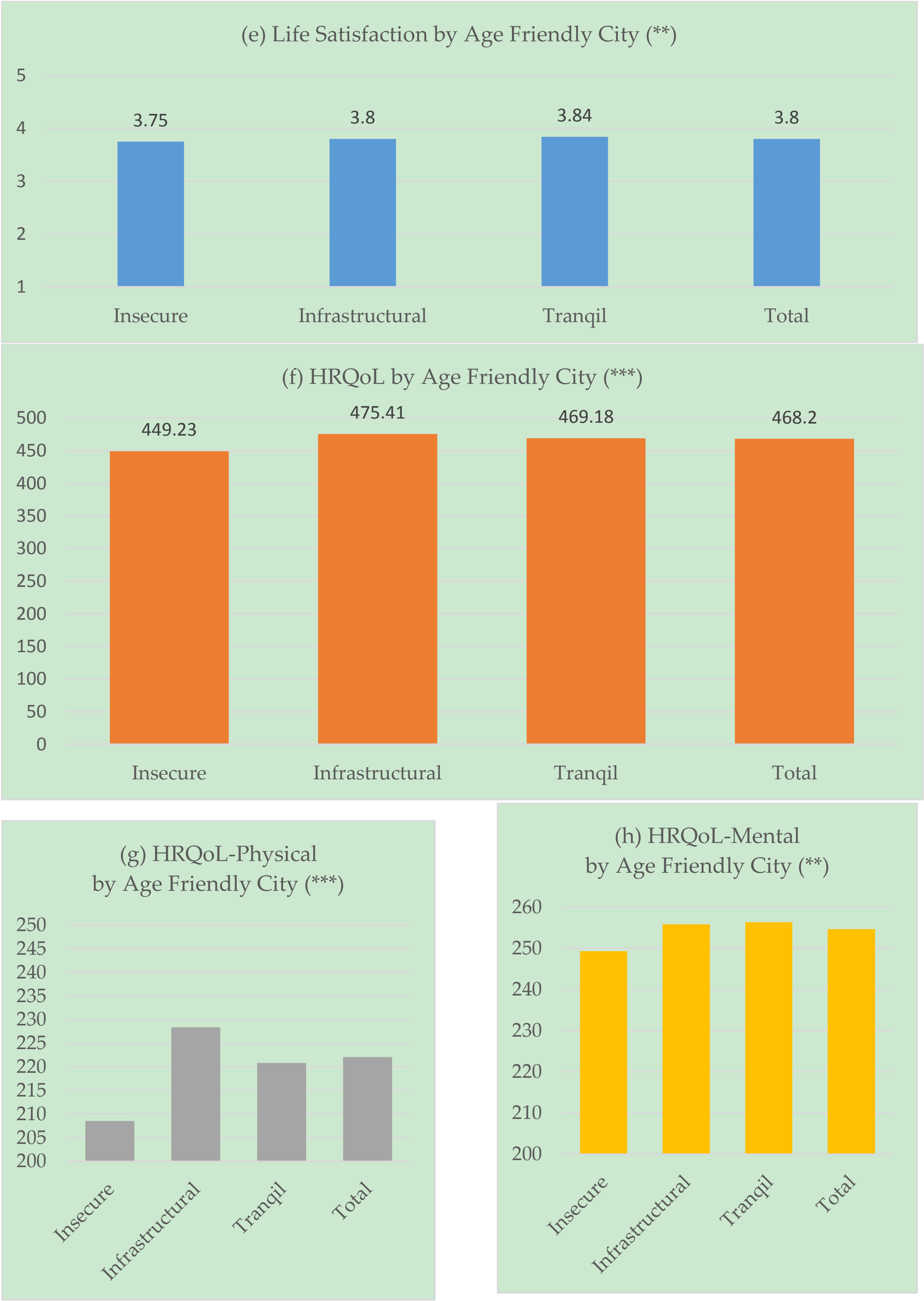

Figure S1. Well-being by active aging and age friendly city clusters. Note: Life satisfaction was analyzed for the sample age 55 and above; quality of life was measured for the sample age 65 and above. Analysis by one-way ANOVA, ** $p < 0.01$, *** $p < 0.001$.

Table S1. The sample size and the backgrounds of the cities in 2017.

Cities	Sample Size	Total Population	Age 55+ %	Age 65+ %	Average Income for the Recipients (NT)	Life Expectancy for Males	Life Expectancy for Females	Higher Education (College/University+ (%)	Primary Industry Occupation (%)
New Taipei	1092	3,986,689	27.7%	12.6%	499,221	78.14	84.27	45.61%	0.69%
Taipei City	807	2,683,257	31.1%	16.4%	634,577	80.82	86.29	60.95%	0.24%
Taoyuan City	510	2,188,017	23.5%	10.8%	507,899	77.73	83.97	45.23%	1.07%
Keelung City	126	371,458	31.1%	14.8%	451,478	76.98	82.92	40.30%	0.39%
Hsinchu City	118	441,132	23.5%	11.5%	646,134	77.94	84.08	53.72%	0.32%
Yilan County	147	456,607	29.9%	15.3%	421,125	76.20	83.59	36.99%	6.58%
Hsinchu County	140	552,169	23.8%	12.0%	681,662	77.08	84.06	47.22%	3.25%
Taichung City	675	2,787,070	24.8%	11.5%	495,874	77.37	83.39	46.29%	2.94%
Miaolee County	161	553,807	29.4%	15.4%	416,082	76.01	82.75	37.91%	6.04%
Changhwa County	369	1,282,458	28.4%	14.8%	389,335	76.47	83.82	37.27%	9.47%
Nantou County	160	501,051	31.8%	16.5%	350,159	75.01	82.41	34.46%	17.57%
Yunlin County	223	690,373	31.5%	17.6%	332,335	74.76	82.63	32.13%	19.70%
Tainan City	560	1,886,522	29.3%	14.4%	431,766	76.67	83.00	42.92%	7.03%
Kaohsiung City	808	2,776,912	29.1%	14.2%	464,956	75.93	82.41	43.65%	3.31%
Chiayi City	121	269,398	27.7%	14.2%	454,919	76.69	83.11	47.85%	1.90%
Chiayi County	172	511,182	33.7%	18.5%	345,739	75.17	83.06	30.56%	21.96%
Pingtung County	266	829,939	31.2%	15.8%	396,335	73.54	80.7	33.85%	16.59%
Taitung County	118	329,237	30.5%	15.3%	399,369	72.77	81.46	36.63%	8.76%
Hwalien County	118	219,540	30.3%	15.6%	413,695	71.50	80.40	29.96%	18.41%
Islands	229	254,409	29.1%	13.5%	---	---	---	---	---
Penghu	---	104,073	29.3%	15.5%	354,109	76.63	84.05	36.84%	5.01%
Kingmen	---	137,456	29.1%	12.2%	---	---	---	48.05%	---
Lienchian	---	12,880	26.8%	10.6%	---	---	---	46.09%	---

Note: The population was based on the statistics in 2016, the same time as the survey year. Some statistic data are unavailable for island areas. Primary industry includes agriculture, forestry, fishing and animal husbandry.

Table S2. Life satisfaction with city and individual factors of older adults (age 55+).

Variables	M1a. City Clusters
**Fixed effects**	
Constant	1.987 (0.115) ***
Age	0.051 (0.007) ***
Sex (male)	−0.072 (0.021) **
Marry (having spouse)	0.017 (0.026)
Education	0.010 (0.002) ***
Self-rated health	0.188 (0.012) ***
Taipei City	−0.080 (0.039) *
Taoyuan City	−0.056 (0.043)
Keelung City	0.055 (0.077)
Hsinchu City	−0.214 (0.086) *
Yilan County	0.013 (0.073)
Hsinchu County	−0.092 (0.076)
Taichung City	−0.058 (0.040)
Miaolee County	0.011 (0.069)
Changhwa County	0.015 (0.050)
Nantou County	−0.108 (0.068)
Yunlin County	0.045 (0.061)
Tainan City	0.077 (0.043)
Kaohsiung City	−0.080 (0.038) *
Chiayi City	−0.269 (0.082) **
Chiayi County	0.137 (0.067) *
Pingtung County	−0.084 (0.05)
Taitung County	0.003 (0.080)
Hwalien County	0.059 (0.079)
Islands	0.008 (0.061)
Work	−0.003 (0.024)
Volunteering	0.030 (0032)
Social groups	−0027 (0.029)
Politics	−0.012 (0.073)
Caregiving	−0.177 (0.036) ***
Physically independent	0.235 (0.098) *
Not depressed	0.281 (0.024) ***
Living alone	−0.181 (0.039) ***
Owning house	0.243 (0.043) ***
Non-poverty	0.057 (0.024) *
Socially connected	0.117 (0.022) ***
Internet	0.065 (0.024) **
Lifelong learning	0.091 (0.044) *
Public transportation: convenient	−0.078 (0.086)
Public transportation: inconvenient	−0.026 (0.087)
Public Transportation: safe	0.133 (0.084)
Respected	0.253 (0.021) ***
**Random effects covariance of cities**	
Residual	0.549 (0.010)
City	0.000 (0.000)
Model fit	−2LL = 13,158.632, BIC = 13,175.952

Note: n = 5816. Missing cases were excluded listwise. Analysis by mixed linear model. Reference groups of categorical variables: Sex (male), marital status (no spouse), city (New Taipei), work (no), volunteering (no), social groups (no), politics (no), caregiving (no), physical function (dependent), non-depressed (depressed), living arrangement (with others), owing house (no), non-poverty (no, i.e. poor), social connected (no), Internet use (no), lifelong learning (no), public transportation convenience (non-users), transportation safety (unsafe), public transportation (non-user), and respected (no). Order variables: Age (1–6), education (1–22), and self-rated health (1–5). −2LL = −2 log likelihood; BIC = Schwar'z Bayesian Criteria.* $p < 0.05$. ** $p < 0.01$, *** $p < 0.001$.

Table S3. Quality of life with cities and individual factors of older adults (aged 65+).

Variables	M2a. HRQoL	M3a. HRQoL-Physical	M4a. HRQoL-Mental
**Fixed effects**			
Constant	187.416 (111.005)	69.900 (54.895)	107.373 (8.349) ***
Age	−11.929 (1.714) ***	−7.323 (0.957) ***	−2.923 (0.529) ***
Sex (male)	8.715 (3.890) *	3.308 (2.171)	2.243 (1.533)
Marry (having spouse)	5.583 (4.533)	3.879 (2.530)	4.509 (1.872) *
Education	−0.008 (0.412)	0.042 (0.230)	0.020 (0.158)
Taipei City	−31.937 (154.959)	−11.426 (76.355)	−14.379 (2.812) ***
Taoyuan City	−29.803 (154.999)	−11.631 (76.380)	−15.347 (3.176) ***
Keelung City	9.605 (155.666)	1.246 (76.646)	8.149 (5.618)
Hsinchu City	−52.856 (155.666)	−29.622 (76.801)	−10.829 (6.295) **
Yilan County	19.366 (155.365)	5.702 (76.712)	13.006 (5.532) *
Hsinchu County	4.018 (155.438)	−4.431 (76.658)	1.234 (5.533)
Taichung City	−14.959 (154.974)	−13.499 (76.364)	−1.111 (2.925)
Miaolee County	−20.130 (155.302)	−9.095 (76.572)	−11.568 (4.997) *
Changhwa County	−5.334 (155.071)	−11.867 (76.426)	4.338 (3.641)
Nantou County	−21.771 (155.282)	−13.817 (76.559)	−4.745 (4.941)
Yunlin County	−32.185 (155.200)	−24.951 (76.508)	−7.782 (4.416)
Tainan City	−7.491 (155.005)	−6.810 (76.384)	2.755 (3.174)
Kaohsiung City	−40.443 (154.963)	−15.702 (76.357)	−19.949 (2.807) ***
Chiayi City	−56.909 (155.536)	−31.514 (76.719)	−22.413 (5.983) ***
Chiayi County	22.192 (155.286)	1.147 (76.561)	16.945 (4.915) **
Pingtung County	−57.972 (15.156)	−28.074 (76.479)	−24.453 (4.178) ***
Taitung County	−68.455 (155.491)	−24.108 (76.691)	−29.079 (5.867) ***
Hwalien County	−45.029 (155.461)	−22.140 (76.671)	−20.398 (45785)***
Islands	−15.198 (155.196)	−7.077 (76.505)	−2.100 (4.505)
Work	31.636 (5.282) ***	18.826 (2.949) ***	11.619 (1.769) ***
Volunteering	28.758 (6.364) ***	14.793 (3.553) ***	11.363 (2.372) ***
Social groups	−3.932 (5.207)	1.980 (2.9076)	−7.166 (2.120) **
Politics	−33.416 (13.606) *	−8.605 (7.595)	−16.423 (5.388) **
Caregiving	−7.958 (7.053)	1.009 (3.937)	−6.403 (2.643) *
Physically independent	258.285 (13.561) ***	152999 (7.570) ***	109.274 (7.101) ***
Happy	33.794 (4.909) ***	12.473 (2.740) ***	21.560 (2.049) ***
Living alone	−7.473 (6.509)	1.130 (3.633)	−8.476 (2.859) **
Owning house	15.760 (8.438)	8.831 (4.710)	7.348 (3.120) *
Non-poverty	−2.229 (4.823)	−2.973 (2.692)	3.712 (1.776) *
Socially connected	30.125 (3.878)	14.517 (2.165) ***	13.714 (1.620) ***
Internet	23.491 (3.878) ***	10.057 (2.667) ***	10.332 (1.789) ***
Lifelong learning	8.475 (8.463)	1.749 (4.724)	4.545 (3.194) ***
Public transportation: convenient	1.016 (15.026)	11.916 (8.387)	−12.412 (6.301) *
Public transportation: inconvenient	−24.180 (15.120)	−6.647 (8.440)	−17.290 (6.382) **
Public Transportation: safe	38.580 (14.619) **	10.472 (8.160)	25.489 (6.158) ***
Respected	11.139 (3.952) **	2.370 (2.207)	7.176 (1.559) ***
**Random effects covariance of cities**			
Residual	10,541.556 (257.687) ***	3284.577 (80.291) ***	2935.754 (54.676) ***
City	11,981.897 (2.748 × 10^{-5})	2907.464 (2.173 × 10^{-5})	0.000 (0.000)
**Model fit**	−2LL = 40,724.004, BIC = 40,740.235	−2LL = 36,821.109, BIC = 36,837.341	−2LL = 62,648.683, BIC = 62,666.003

Note: n = 3395. Missing cases were excluded listwise. Analysis by mixed linear model. Reference groups of categorical variables: Sex (male), marital status (no spouse), city (New Taipei), work (no), volunteering (no), social groups (no), politics (no), caregiving (no), physical function (dependent), mental well-being (not happy), living arrangement (with others), owing house (no), non-poverty (no, i.e. poor), social connected (no), Internet use (no), lifelong learning (no), public transportation convenience (non-users), transportation safety (unsafe), public transportation (non-user), and respected (no). Order variables: Age (1–6), education (1–22). −2LL = −2 log likelihood; BIC = Schwar'z Bayesian Criteria.* $p < 0.05$. ** $p < 0.01$, *** $p < 0.001$.

Table S4. Association of active aging city typologies with individuals' age, sex, and health by multinomial logistic regression (odds ratios).

Variables	Content City Cluster	Developed City Cluster	Participatory City Cluster
Sex: female	0.877	0.859	0.762
Age 65–74	0.498	0.463	0.511
Age 75+	1.442	1.370	0.863
Self-rated health	1.077	1.408	1.062
Age 65–74 * self-rated health	1.202	1.247	1.185
Age 65+ * self-rated health	0.836	0.878	0.992
Model fit: −2 log likelihood = 447.672, Chi-square = 64.285, df = 18, $p < 0.001$.			

Note: The reference group: Active Aging City cluster (pioneer), sex (male), age (age 55–74). All the parameters were not significant.

Table S5. Association of age friendly city typologies with individuals' age, sex, and health by multinomial logistic regression (odds ratios).

Variables	Infrastructural City Cluster	Tranquil City Cluster
Sex: female	0.984	0.944
Age 65–74	0.849	0.685
Age 75+	1.406	2.470 **
Self-rated health	1.277 ***	1.235 **
Age 65–74 * self-rated health	1.082	1.137
Age 75+ * self-rated health	0.890	0.776 **
Model fit: −2 log likelihood = 381.163, Chi-square = 68.275, df = 12, $p < 0.001$.		

Note: The reference group: Age Friendly City cluster (Insecure), sex (male), age (age 55-64). * $p < 0.05$, ** $p < 0.01$, *** $p < 0.001$.
